# 4,4′-[*p*-Phenyl­enebis(­oxy)]dibutanoic acid

**DOI:** 10.1107/S1600536811036828

**Published:** 2011-09-17

**Authors:** Zhi Li

**Affiliations:** a Department of Chemistry, School of Science, Beijing Jiaotong University, No. 3 Shang Yuan Cun Road, HaiDian District 100044, People’s Republic of China

## Abstract

The complete mol­ecule of the title compound, C_14_H_18_O_6_, has a center of inversion at the centroid of the benzene ring and the asymmetric unit contains one half-mol­ecule. The conformation of the side chain is *anti* [C—C—C—C = −171.40 (17)°]. In the crystal, pairs of head-to-head carb­oxy­lic acid O—H⋯O hydrogen bonds link the mol­ecules into infinite zigzag chains propagating along [130]. Weak C—H⋯π inter­actions between adjacent chains expand the structure into a layered network in the *ac* plane.

## Related literature

For general background to phen­oxy­acetic acid derivatives, see: Yada (1959 ▶); Zheng *et al.* (2007 ▶); Deng *et al.* (2010 ▶); Xiong *et al.* (2010 ▶); Fu *et al.* (2011 ▶). For related structures of multidentate *O*-donor ligands such as benzene-1,4-di­oxy­diacetic acid and benzene-1,4-di­oxy­dibutanoic acid, see: Dai *et al.* (2009 ▶); Zhu *et al.* (2008 ▶); Li *et al.* (2010 ▶); Yang *et al.* (2010 ▶); Zhao (2011 ▶). For the synthesis of the title compound, see: Zhang *et al.* (2009 ▶). For standard bond lengths, see: Allen *et al.* (1987 ▶).
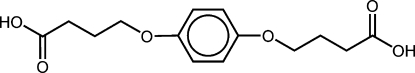


## Experimental

### 

#### Crystal data

C_14_H_18_O_6_
*M* *_r_* = 282.28
Triclinic, 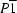

*a* = 4.8389 (11) Å
*b* = 6.6300 (15) Å
*c* = 11.406 (3) Å
α = 83.067 (5)°
β = 81.249 (5)°
γ = 71.095 (4)°
*V* = 341.16 (13) Å^3^
*Z* = 1
Mo *K*α radiation
μ = 0.11 mm^−1^
*T* = 296 K
0.21 × 0.19 × 0.18 mm

#### Data collection

Bruker SMART CCD diffractometer
Absorption correction: multi-scan (*SADABS*; Sheldrick, 1996 ▶) *T*_min_ = 0.978, *T*_max_ = 0.981
1861 measured reflections
1170 independent reflections
1025 reflections with *I* > 2σ(*I*)
*R*_int_ = 0.023

#### Refinement

*R*[*F* ^2^ > 2σ(*F* ^2^)] = 0.050
*wR*(*F* ^2^) = 0.162
*S* = 1.02
1170 reflections
92 parameters
H-atom parameters constrained
Δρ_max_ = 0.30 e Å^−3^
Δρ_min_ = −0.20 e Å^−3^

### 

Data collection: *SMART* (Bruker, 2007 ▶); cell refinement: *SAINT* (Bruker, 2007 ▶); data reduction: *SAINT*; program(s) used to solve structure: *SHELXS97* (Sheldrick, 2008 ▶); program(s) used to refine structure: *SHELXL97* (Sheldrick, 2008 ▶); molecular graphics: *SHELXTL* (Sheldrick, 2008 ▶); software used to prepare material for publication: *SHELXTL*.

## Supplementary Material

Crystal structure: contains datablock(s) global, I. DOI: 10.1107/S1600536811036828/hb6398sup1.cif

Structure factors: contains datablock(s) I. DOI: 10.1107/S1600536811036828/hb6398Isup2.hkl

Supplementary material file. DOI: 10.1107/S1600536811036828/hb6398Isup3.cml

Additional supplementary materials:  crystallographic information; 3D view; checkCIF report

## Figures and Tables

**Table 1:** Hydrogen-bond geometry (Å, °) *Cg*1 is the centroid of the C5–C7/C5′–C7′ ring.

*D*—H⋯*A*	*D*—H	H⋯*A*	*D*⋯*A*	*D*—H⋯*A*
O2—H2⋯O1^i^	0.82	1.85	2.668 (3)	174
C4—H4*B*⋯*Cg*1^ii^	0.97	2.89	3.703 (3)	142

Symmetry codes: (i) 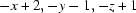; (ii) 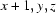.

**Fig. 1.**
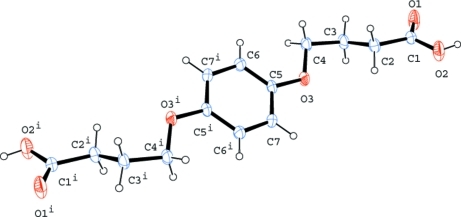
The molecular structure of the title compound, with displacement ellipsoids at the 30% probability level. Symmetry code: (i) -x, 1 - y, -z.

**Fig. 2.**
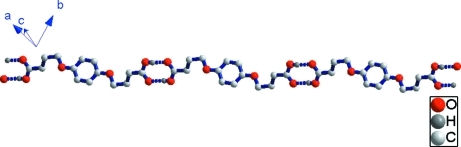
Part of the zigzag infinite chain structure of the title compound, linked via hydrogen bonds (dashed lines) lying in the [1 3 0] direction. H atoms have been omitted for clarity, except for those involved in hydrogen-bonded interactions.

**Fig. 3.**
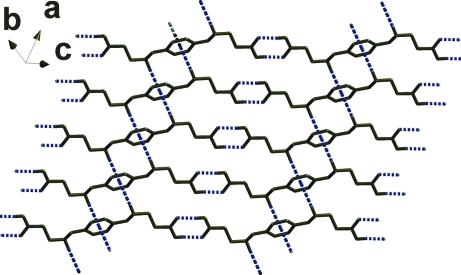
Part of 2 d the crystal structure showing hydrogen bonds and C—H···π contants as dashed lines. H atoms, except for those involved in hydrogen bonds, are not included.

## supplementary crystallographic information

### Comment

Compounds of the phenoxyacetic acid and their derivatives have good herbicidal activity and become excellent plant growth regulators (Yada, 1959; Zheng *et al.*, 2007; Deng *et al.*, 2010; Xiong *et al.*, 2010; Fu *et al.*, 2011). Also, the two phenoxyacetate moieties have versatile flexiable bonding fashions to metal ions and easily forms coordination polymers (Dai *et al.*, 2009; Zhu *et al.*, 2008; Li *et al.*, 2010; Yang *et al.*, 2010; Zhao *et al.*, 2011). Benzene-1,4-dioxydibutanoic acid is an interesting dicarboxylate ligand and its cobalt polymer has been reported by Zhao *et al.* 2011. To further investigate this family of ligands, the title compound, (I), was synthesized and its structure was confirmed by X-ray diffraction. X-ray diffraction analysis reveals that the asymmetric unit of the title compound contains one half-molecule and has a crystallographic inversion center at the centroid of the benzene ring (Fig. 1). The benzene-connected portions of the alkoxy substituents lie almost coplanar with the C3–C4–O3–C5 torsion angle of 176.81 (16)°. In the molecule of (I) (Fig. 1) the bond lengths are within normal ranges (Allen *et al.*, 1987). The C1—O2, C4—O3 and C5—O3 bond length of 1.287 (3), 1.428 (2) and 1.375 (2) Å, respectively, indicate the presence of typical single bonds. Whereas the C1–O1 [1.221 (3) Å] bond lengths correspond to a typical C═O bond.

In the crystal structure, it is noteworthy that pairs of intermolecular O—H···O hydrogen bonds link head-to-tail the molecules into infinite 1 d chains along the [1 3 0] direction (Fig. 2). Neighboring 1 d chains are in turn interacting with each other through C—H···π stacking interactions with the H···π distances of 2.89 (3) Å to form infinite stacks along *b* axis, thus leading to an interwoven two dimensional network held together by O—H···O interactions and C—H···π stacking (Fig. 3).

### Experimental

Reagents and solvents were of commercially available quality. The title compound was synthesized according to the method of Zhang *et al.* 2009. To a solution of *p*-dihydroxybenzene (0.01 mol) in acetonitrile (50 ml), anhydrous potassium carbonate (0.02 mol) and ethyl 4-bromobutanoate (0.01 mol) were mixed. The mixture solution was refluxed for 6 h and filtered. The filtrate was evaporated under reduced pressure and the solid product was dissolved in water/ethanol (1:2 *v*/*v*), then sodium hydroxide (0.02 mol) was added. The solution was refluxed for another 24 h, then acidified with dilute HCl. The crude product was separated by filtration and crystals of the title compound were prepared by recrystallization from a mixture of water and ethanol (1:1 *v*/*v*).

### Refinement

All H atoms were placed in idealized positions (C—H = 0.93–0.97 Å, O—H = 0.82 Å and refined as riding atoms with *U*_iso_(H) = 1.2*U*_eq_(C) and with *U*_iso_(H) = 1.5*U*_eq_(O).

### Crystal data

**Table:**

C_14_H_18_O_6_	*Z* = 1
*M**_r_* = 282.28	*F*(000) = 150
Triclinic, *P*1	*D*_x_ = 1.374 Mg m^−^^3^
Hall symbol: -P 1	Mo *K*α radiation, λ = 0.71073 Å
*a* = 4.8389 (11) Å	Cell parameters from 1172 reflections
*b* = 6.6300 (15) Å	θ = 3.3–29.3°
*c* = 11.406 (3) Å	µ = 0.11 mm^−^^1^
α = 83.067 (5)°	*T* = 296 K
β = 81.249 (5)°	Block, colorless
γ = 71.095 (4)°	0.21 × 0.19 × 0.18 mm
*V* = 341.16 (13) Å^3^	

### Data collection

**Table:**

Bruker SMART CCD diffractometer	1170 independent reflections
Radiation source: fine-focus sealed tube	1025 reflections with *I* > 2σ(*I*)
graphite	*R*_int_ = 0.023
φ and ω scans	θ_max_ = 25.0°, θ_min_ = 1.8°
Absorption correction: multi-scan (*SADABS*; Sheldrick, 1996)	*h* = −5→5
*T*_min_ = 0.978, *T*_max_ = 0.981	*k* = −6→7
1861 measured reflections	*l* = −13→11

### Refinement

**Table:**

Refinement on *F*^2^	Primary atom site location: structure-invariant direct methods
Least-squares matrix: full	Secondary atom site location: difference Fourier map
*R*[*F*^2^ > 2σ(*F*^2^)] = 0.050	Hydrogen site location: inferred from neighbouring sites
*wR*(*F*^2^) = 0.162	H-atom parameters constrained
*S* = 1.02	*w* = 1/[σ^2^(*F*_o_^2^) + (0.0972*P*)^2^ + 0.1511*P*] where *P* = (*F*_o_^2^ + 2*F*_c_^2^)/3
1170 reflections	(Δ/σ)_max_ < 0.001
92 parameters	Δρ_max_ = 0.30 e Å^−^^3^
0 restraints	Δρ_min_ = −0.20 e Å^−^^3^

### Special details

**Table:**

Geometry. All e.s.d.'s (except the e.s.d. in the dihedral angle between two l.s. planes) are estimated using the full covariance matrix. The cell e.s.d.'s are taken into account individually in the estimation of e.s.d.'s in distances, angles and torsion angles; correlations between e.s.d.'s in cell parameters are only used when they are defined by crystal symmetry. An approximate (isotropic) treatment of cell e.s.d.'s is used for estimating e.s.d.'s involving l.s. planes.
Refinement. Refinement of *F*^2^ against ALL reflections. The weighted *R*-factor *wR* and goodness of fit *S* are based on *F*^2^, conventional *R*-factors *R* are based on *F*, with *F* set to zero for negative *F*^2^. The threshold expression of *F*^2^ > 2σ(*F*^2^) is used only for calculating *R*-factors(gt) *etc*. and is not relevant to the choice of reflections for refinement. *R*-factors based on *F*^2^ are statistically about twice as large as those based on *F*, and *R*- factors based on ALL data will be even larger.

### Fractional atomic coordinates and isotropic or equivalent isotropic displacement parameters (Å^2^)

**Table:**

	*x*	*y*	*z*	*U*_iso_*/*U*_eq_	
O1	0.9691 (4)	−0.2476 (3)	0.45159 (18)	0.0672 (6)	
O2	0.7156 (4)	−0.4427 (3)	0.40552 (18)	0.0663 (6)	
H2	0.8171	−0.5317	0.4509	0.099*	
O3	0.4183 (3)	0.2151 (2)	0.13043 (12)	0.0412 (5)	
C1	0.7769 (4)	−0.2666 (3)	0.39978 (17)	0.0364 (5)	
C2	0.5900 (4)	−0.0856 (3)	0.32641 (18)	0.0410 (6)	
H2A	0.4016	−0.0255	0.3730	0.049*	
H2B	0.5536	−0.1415	0.2575	0.049*	
C3	0.7239 (5)	0.0911 (3)	0.28431 (19)	0.0415 (6)	
H3A	0.7874	0.1333	0.3516	0.050*	
H3B	0.8965	0.0371	0.2276	0.050*	
C4	0.5123 (5)	0.2853 (3)	0.22662 (18)	0.0409 (5)	
H4A	0.3448	0.3480	0.2837	0.049*	
H4B	0.6090	0.3920	0.1973	0.049*	
C5	0.2114 (4)	0.3636 (3)	0.06782 (16)	0.0328 (5)	
C6	0.1047 (4)	0.5809 (3)	0.08497 (18)	0.0375 (5)	
H6	0.1744	0.6355	0.1417	0.045*	
C7	0.1059 (4)	0.2846 (3)	−0.01715 (17)	0.0373 (5)	
H7	0.1775	0.1394	−0.0288	0.045*	

### Atomic displacement parameters (Å^2^)

**Table:**

	*U*^11^	*U*^22^	*U*^33^	*U*^12^	*U*^13^	*U*^23^
O1	0.0781 (12)	0.0427 (10)	0.0919 (14)	−0.0226 (9)	−0.0584 (11)	0.0239 (9)
O2	0.0855 (14)	0.0421 (10)	0.0817 (13)	−0.0260 (9)	−0.0510 (11)	0.0240 (8)
O3	0.0462 (9)	0.0296 (8)	0.0429 (8)	−0.0002 (6)	−0.0218 (6)	0.0041 (6)
C1	0.0388 (10)	0.0319 (11)	0.0358 (10)	−0.0068 (8)	−0.0098 (8)	0.0033 (8)
C2	0.0387 (11)	0.0388 (12)	0.0417 (11)	−0.0058 (9)	−0.0149 (9)	0.0063 (9)
C3	0.0424 (11)	0.0360 (11)	0.0446 (12)	−0.0072 (9)	−0.0207 (9)	0.0086 (9)
C4	0.0460 (11)	0.0323 (11)	0.0431 (11)	−0.0073 (9)	−0.0192 (9)	0.0056 (8)
C5	0.0313 (9)	0.0295 (10)	0.0334 (10)	−0.0044 (8)	−0.0095 (7)	0.0065 (7)
C6	0.0420 (11)	0.0323 (11)	0.0377 (10)	−0.0075 (8)	−0.0140 (8)	−0.0002 (8)
C7	0.0428 (11)	0.0252 (9)	0.0394 (11)	−0.0032 (8)	−0.0109 (8)	0.0016 (7)

### Geometric parameters (Å, °)

**Table:**

O1—C1	1.221 (3)	C3—H3A	0.9700
O2—C1	1.287 (3)	C3—H3B	0.9700
O2—H2	0.8200	C4—H4A	0.9700
O3—C5	1.375 (2)	C4—H4B	0.9700
O3—C4	1.428 (2)	C5—C7	1.386 (3)
C1—C2	1.498 (3)	C5—C6	1.391 (3)
C2—C3	1.512 (3)	C6—C7^i^	1.385 (3)
C2—H2A	0.9700	C6—H6	0.9300
C2—H2B	0.9700	C7—C6^i^	1.385 (3)
C3—C4	1.512 (3)	C7—H7	0.9300
			
C1—O2—H2	109.5	H3A—C3—H3B	107.8
C5—O3—C4	117.68 (15)	O3—C4—C3	107.15 (16)
O1—C1—O2	122.66 (18)	O3—C4—H4A	110.3
O1—C1—C2	122.65 (18)	C3—C4—H4A	110.3
O2—C1—C2	114.68 (18)	O3—C4—H4B	110.3
C1—C2—C3	114.15 (16)	C3—C4—H4B	110.3
C1—C2—H2A	108.7	H4A—C4—H4B	108.5
C3—C2—H2A	108.7	O3—C5—C7	115.72 (16)
C1—C2—H2B	108.7	O3—C5—C6	124.72 (18)
C3—C2—H2B	108.7	C7—C5—C6	119.56 (18)
H2A—C2—H2B	107.6	C7^i^—C6—C5	119.61 (19)
C4—C3—C2	112.76 (16)	C7^i^—C6—H6	120.2
C4—C3—H3A	109.0	C5—C6—H6	120.2
C2—C3—H3A	109.0	C6^i^—C7—C5	120.83 (18)
C4—C3—H3B	109.0	C6^i^—C7—H7	119.6
C2—C3—H3B	109.0	C5—C7—H7	119.6
			
O1—C1—C2—C3	21.4 (3)	C4—O3—C5—C6	5.5 (3)
O2—C1—C2—C3	−159.7 (2)	O3—C5—C6—C7^i^	−179.46 (17)
C1—C2—C3—C4	−171.40 (17)	C7—C5—C6—C7^i^	0.1 (3)
C5—O3—C4—C3	176.81 (16)	O3—C5—C7—C6^i^	179.49 (16)
C2—C3—C4—O3	−57.1 (2)	C6—C5—C7—C6^i^	−0.1 (3)
C4—O3—C5—C7	−174.06 (17)		

Symmetry codes: (i) −*x*, −*y*+1, −*z*.

### Hydrogen-bond geometry (Å, °)

**Table:**

Cg1 is the centroid of the C5–C7/C5'–C7' ring.

**Table:**

*D*—H···*A*	*D*—H	H···*A*	*D*···*A*	*D*—H···*A*
O2—H2···O1^ii^	0.82	1.85	2.668 (3)	174
C4—H4B···Cg1^iii^	0.97	2.89	3.703 (3)	142

Symmetry codes: (ii) −*x*+2, −*y*−1, −*z*+1; (iii) *x*+1, *y*, *z*.
